# Pharmaceutical Analysis of Protein–Peptide Coformulations and the Influence of Polysorbates

**DOI:** 10.1021/acs.molpharmaceut.5c00119

**Published:** 2025-04-30

**Authors:** Joseph Whiteley, Susanna Abrahmsén-Alami, Jonathan Booth, Steve Mellor, James Humphrey, Laura J. Waters

**Affiliations:** † School of Applied Sciences, University of Huddersfield, Queensgate, Huddersfield HD1 3DH, U.K.; ‡ Sustainable Innovation & Transformational Excellence, Pharmaceutical Technology & Development, Operations, AstraZeneca, Gothenburg SE-431 83, Sweden; § New Modalities & Parenterals Development, Pharmaceutical Technology & Development, Operations, AstraZeneca, Charter Way, Macclesfield SK10 2NA, U.K.; ∥ Croda Europe Ltd, Cowick Hall, Snaith, Goole DN14 9AA, U.K.

**Keywords:** binding affinity, coformulations, DSC, ITC, thermal stability

## Abstract

Coformulation is an approach to formulating multiple biopharmaceutical therapeutics in a single formulation, promising the benefits of both therapies in one dose. However, as molecular stability is a key consideration in traditional biopharmaceutical formulations, stability of coformulations will require extensive investigation. This study evaluated the effects of traditional formulation stabilizers, specifically surfactants, at different grades, namely, regular grade (RG) Tween 20 and Tween 80 and Super-refined Polysorbate 20 and 80. Their effects were assessed through their interactions with human serum albumin (HSA) and a glucagon-like peptide-1 (GLP-1) receptor agonist (MEDI7219). Isothermal titration calorimetry (ITC) and differential scanning calorimetry (DSC) were implemented to determine the strength of the binding interactions and thermal stability of the tertiary system. ITC confirmed that upon titration of MEDI7219 into a solution of HSA and RG, Tween 20 the binding affinity of the peptide was reduced, resulting in negatively cooperative binding. However, when the peptide was titrated into a solution of HSA and both grades of Tween 80, the binding affinity increased with positive cooperative binding. DSC established that MEDI7219 increased the thermal stability of HSA to a similar extent to the polysorbates. Combining peptide and polysorbate did not further increase the thermal stability of HSA; however, it did reduce the unfolding of HSA molecules in the absence of heat. Overall, the unique findings in this study have demonstrated that the order of addition in a ternary coformulation affects the final composition which is an important consideration for pharmaceutical development.

## Graphical abstract

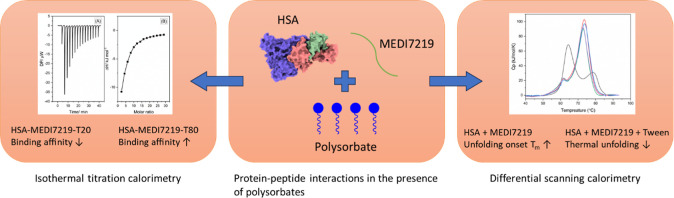

## 1 Introduction

Biopharmaceuticals or biologics are an ever growing segment of the pharmaceutical industry, with over 300 products entering the market over the last 40 years.^1^ The field of biologics consists of many types of therapeutic products, including monoclonal antibodies, vaccines and peptides, with a particular focus within the field of oncology.^2^ With new therapies constantly in development, it is paramount that research into biologic formulation is being pursued to help take new therapies from discovery to production as efficiently as possible. Coformulations are an area within biologic treatment thought to give improved therapeutic benefit when compared with biologic formulations containing one protein.^3^ Such improvements include formulating multiple medications into a single multidose coformulation to improve patient compliance.^3^ However, even in single-dose biologics, protein stability has been an area of investigation for decades, and many published papers and reviews have investigated this topic.^4^ The interest in this area derives from the fact that proteins are fragile, and once they have begun to denature they can cause undesired effects that trigger activation of the immune system.^5^ In order to stabilize proteins, it is common practice to add excipients to formulations,^6^ most notably surfactants.^7,8^ Surfactants, such as polysorbates, stabilize proteins via interfacial interactions and outcompete protein molecules for binding sites at an interface, preventing protein aggregation.^8^ The extent of stabilization depends on different factors including the type of polysorbate, for example, Tween 20 (T20) or Tween 80 (T80) and their level of purity, regular grade (RG) or Super-refined (SR). This has recently been investigated using transport and globular proteins.^9^ While polysorbate effects on single protein formulations have substantial evidence through publication, excipient effects on coformulations have not been well investigated.

To assess protein–polysorbate interactions, various analytical techniques can be employed, for example, X-ray scattering,^10^ circular dichroism,^11^ and transmission electron microscopy^12^ but in order to quantify the binding interactions of each ligand independently, isothermal titration calorimetry (ITC) is typically used.^13^ ITC is usually applied to characterize the thermodynamics of binding events between a macromolecule and a ligand.^14^ However, in coformulations, there are two ligands present (peptide and surfactant) that can themselves interact and one ligand’s binding interactions could influence the other. First, ligand 1 and 2 could behave competitively; one ligand could prevent the binding of the other. Second, the ligands could bind noncompetitively; one ligand does not influence the binding of the other. Finally, one ligand could bind allosterically to the macromolecule and change the binding interactions between the macromolecule and the other ligand. Deconvoluting these combinations of interactions is a challenge that has not often been attempted. There is however a study conducted by Velazquez-Campoy and co-workers that proposed a system that allows resolution of multiple ligand binding interactions using the reaction pathway seen in Figure 1.^15^

**Figure 1:**
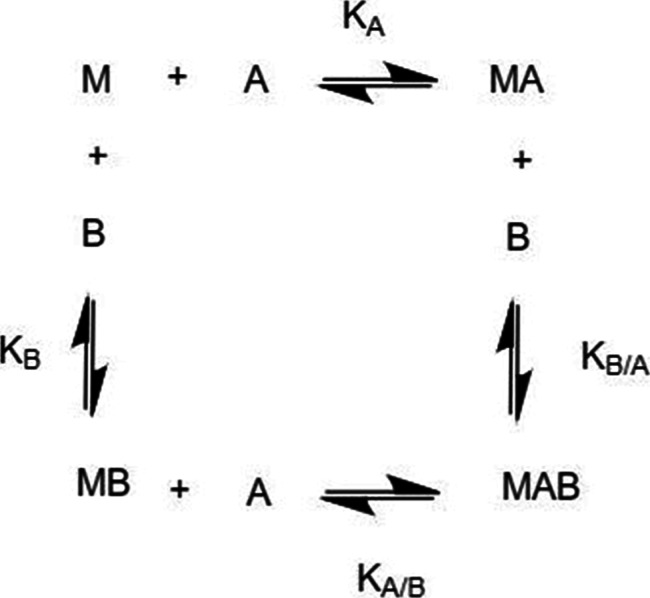
General reaction scheme for the interaction of a macromolecule, M with two ligands, A and B. Adapted from Velazquez-Campoy and co-workers.^15^

As Figure 1 demonstrates, the overall reaction results in the same product (MAB), regardless of which pathway is pursued first; therefore, the pathways can be compared. The distinction between the pathways is with regard to the reaction constants *K*_A_ and *K*_B/A_ as well as *K*_B_ and *K*_A/B_. In the case of a cooperative binding interaction, the binding of ligand A to the macromolecule, *M*, changes the binding of the macromolecule to ligand B. If the two pathways result in the same product, Velazquez-Campoy and co-workers explain this by defining a constant, α^15^ (Figure 2).

**Figure 2:**
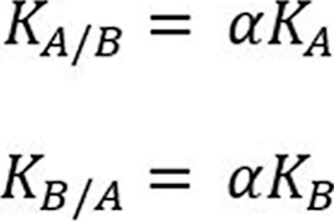
Individual binding constants of one reaction pathway are different to the other (yet equivalent in their total binding constant values) resulting in a reciprocal modification of the binding interactions with the macromolecule.

For this study, a ternary system, as displayed in Figure 3, was studied that enabled the analysis of the binding interactions of a pharmaceutically relevant peptide (MEDI7219) and a model protein [human serum albumin (HSA)] in the presence and absence of polysorbates.

**Figure 3:**
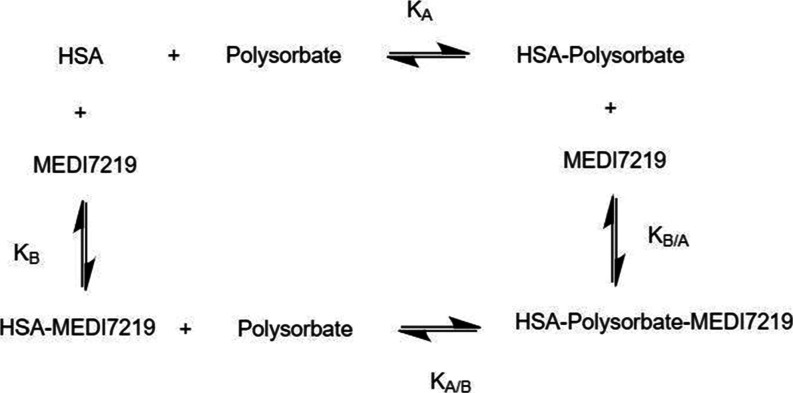
Reaction pathway for a model protein (HSA) interacting with a pharmaceutically relevant peptide (MEDI7219) in the presence of polysorbate to create a ternary species.

Following ITC, the second technique considered is differential scanning calorimetry (DSC). DSC is often used to assess thermal stability and long-term storage for drug formulations.^16,17^ In this multicomponent system, analysis involved heating HSA is the presence of MEDI7219 and polysorbates individually and comparing this with the profiles produced when heated together. DSC profiles indicate the temperature at which a protein undergoes thermal denaturation and therefore are indicative of any changes in thermal stability. This study investigates the influence of two different grades of polysorbate 20 and 80 [regular (RG) and superfine (SR) grades] using a combination of ITC and DSC to give a full thermodynamic description of the binding interactions and their effect on the stability of protein–peptide–polysorbate interactions.

## 2 Experimental Section

### 2.1 Materials

Polyoxyethylene (20) sorbitan monolaurate (“polysorbate 20”) and polyoxyethylene (20) sorbitan monooleate (“polysorbate 80”) were donated by Croda Europe Ltd.: standard compendial grades, referred to as Tween 20 (BN:50702) and Tween 80 (BN:49659A), and high purity grades, referred to as Super Refined Polysorbate 20 (BN:0001814116) and Super Refined Polysorbate 80 (BN:0001779440). The Super Refined versions are distinct from the standard grades through their chemical composition including a low peroxide value (2.0 mequiv O_2_/kg max.), limited formaldehyde (10 ppm max.), low residual EO (ethylene oxide) (1 ppm max.), low 1,4-dioxane (5 ppm max.), low residual Na and K (5 ppm max.), low moisture (0.2% max.), and decreased cellular irritation and microbial testing. A glucagon-like peptide-1 receptor agonist lipidated peptide, MEDI7219 (purity 97.5%), was provided by AstraZeneca as a lyophilized powder. The protein, albumin from human serum (HSA) (>96%), was purchased from Sigma-Aldrich UK as a lyophilized powder. This protein was chosen as binding activity toward MEDI7219 had previously been demonstrated.^18^ Potassium phosphate saline buffer (pH 7.4) was composed of 1.8 mM KH_2_PO_4_ (>99%, Sigma-Aldrich, UK), 8.2 mM K_2_HPO_4_ (>98%, Sigma-Aldrich, UK), 2.7 mM KCl (>99%, Fisher Scientific, UK), 140 mM NaCl (99.5%, Acros Organics, UK), and ultrapure water (18.2 MΩ·cm).

### 2.2 Methods

#### 2.2.1 ITC

Lyophilized protein powders were rehydrated by adding buffer to the protein to achieve a concentration of 10 mg/mL, then stored at 5 °C for 72 h or until fully dissolved. Polysorbate concentrations chosen were 0.1× and 1× the critical micelle concentration (CMC) of each respective polysorbate.^19^ Due to the dilution of injection by the ITC, polysorbate formulations were formulated at 10 times the desired concentration. ITC experiments were performed using a Microcal PEAQ-ITC instrument from Malvern Panalytical. Prior to each experiment, the sample cell and syringe were cleaned using 20% Contrad 70 detergent, rinsed with ultrapure water, and dried using methanol. Titration experiments were conducted at 30 °C to ensure that the experimental temperature was above laboratory ambient temperature. As the Microcal PEAQ-ITC lacks a cooling unit, it was necessary to conduct experiments at a temperature that could be maintained consistently. The reference cell was filled with ultrapure water for all experiments. The 200 μL sample cell was filled with protein solution, and the 40 μL injection syringe was filled with polysorbate solution and equilibrated at 5 °C below the experimental temperature. The reference power was set to 41.86 μJ/s (10 μcal/s). Experiments comprised 14 injections of 2.5 μL at an injection speed of 0.5 μL/s. The time between injections was set long enough to allow the heat signals to return to the baseline before the next injection. The solution in the reaction cell was continuously stirred at a speed of 500 rpm. Experiments requiring the titration of peptide or surfactant into protein–surfactant or protein–peptide required the continuation of one experiment into the next, to maintain precise concentration ratios between experimental pathways.^15^ As a result, once an experiment had concluded, excess solution was withdrawn and the next titration was conducted into the resulting solution. A reference experiment of polysorbate injected into buffer solution was conducted under identical conditions and subtracted from each run that contained polysorbate in the injection syringe. For experiments that contained MEDI7219 in the injection syringe, a control experiment could not be subtracted in the same manner as the polysorbate experiments. This was suspected to be a consequence of limitations in the software and the interactions generating small binding heats, leading to a noisier thermogram. However, a fitted offset was used instead, which reduced noise and allowed a smooth heat integration. Data were analyzed using MicroCal PEAQ-ITC Analysis Software v1.41. The binding model was analyzed using the one set of binding sites software preset, resulting in the values of changes in the Gibbs free energy (Δ*G*), enthalpy (Δ*H*), and entropy in relation to temperature (−*T*Δ*S*). Each experiment was repeated a minimum of three times to assess the reproducibility.

#### 2.2.2 DSC

Lyophilized protein and MEDI7219 were rehydrated by adding buffer to achieve a concentration of 30 mg/mL, then stored at 5 °C for 72 h or until fully dissolved. Polysorbate solutions were prepared at 3 times higher than the desired concentration to account for dilution in the formulation after mixing with MEDI7219. Polysorbate concentrations in experiments were 0.1× and 1× each polysorbates respective CMC.^19^ Samples were produced by adding equal volumes of each component to provide the desired formulation. DSC experiments were conducted using a Microcal PEAQ-DSC instrument from Malvern Panalytical. Prior to each experiment, DSC cells were washed with 2% Decon 90 detergent and then rinsed with ultrapure water. The DSC capillary cell volume was 250 μL for both reference and sample cells; for each experiment, the reference cell contained buffer solution. The sample cell contained the prepared formulation of 10 mg/mL HSA, 10 mg/mL MEDI7219, and the polysorbate concentration being investigated. The scanning temperature range was set at 20–105 °C at a rate of 60 °C/h with a total scan time of 85 min and repeated in triplicate. A reference scan of the buffer solution was conducted under identical conditions and subtracted from each run. Data were analyzed using MicroCal PEAQ-DSC Analysis Software v1.64.

## 3 Results and Discussion

### 3.1 ITC

Typically, in ITC, a ligand is titrated into a macromolecule, and the two molecules undergo a binding event characterized by binding energies by measuring the energy output or input to maintain a constant temperature. In this investigation, however, two ligand molecules were being investigated. First, when considering the influence of polysorbates on MEDI7219–HSA binding interactions, it was necessary to perform two titrations. The first titration was MEDI7219 into HSA, an example of which is displayed in Figure 4 with an endothermic interaction and association constant of 2.4 × 10^4^ M^–1^. This value was significantly lower than the association constant of native GLP-1 which has been reported to be 2.3 × 10^8^ M^–1^ ^20^ but higher than other modified forms of GLP-1.^21^ This increase in the association constant is most likely due to the modification of MEDI7219 when compared with native GLP-1 including bis-lipidation and amino acid substitutions.^18^ Data were analyzed using the aforementioned software, and Δ*G* for this interaction was −25.5 kJ mol^–1^, confirming the spontaneity of this binding interaction.

**Figure 4:**
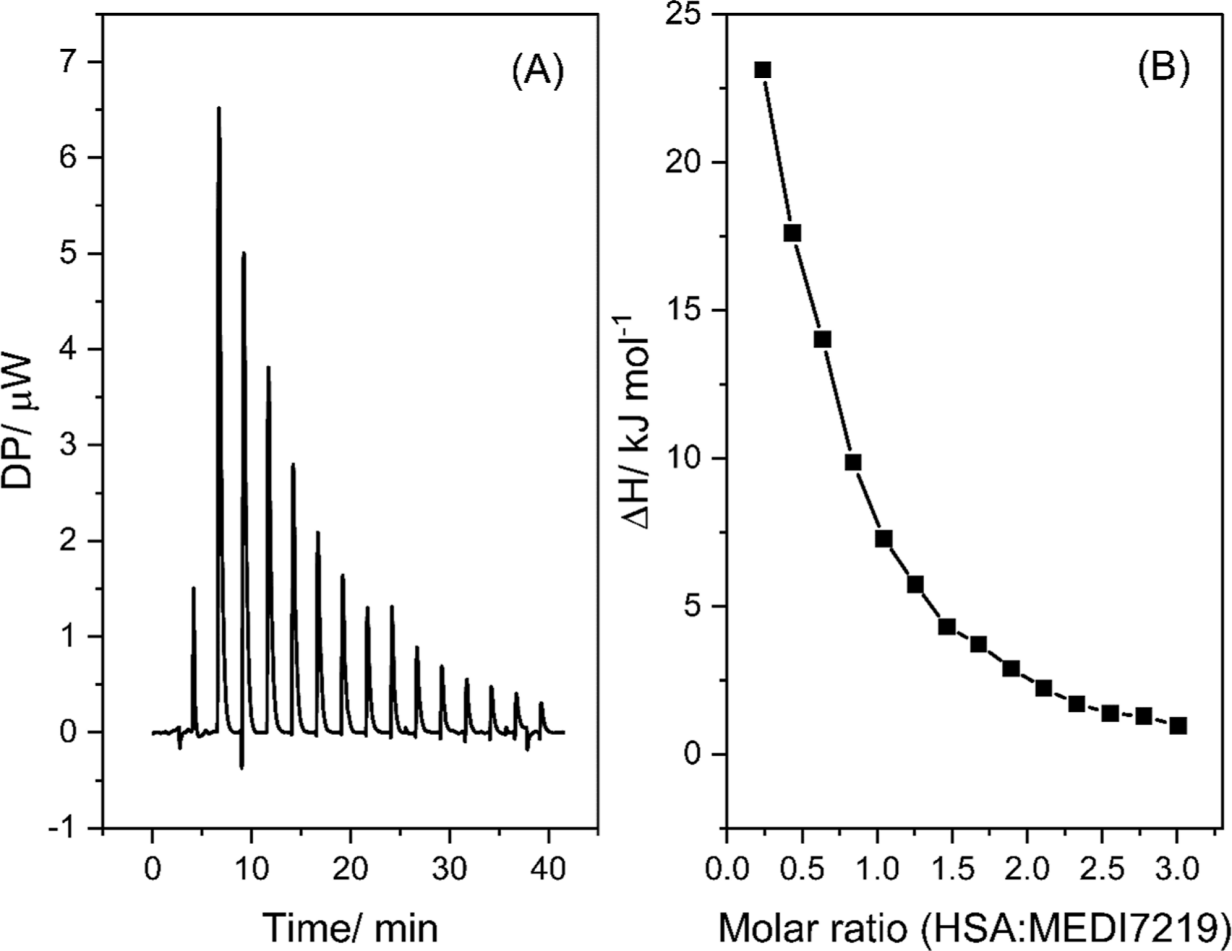
Example of an ITC thermogram of the interaction between MEDI7219 and HSA at 30 °C. Heat flow signal (A). Integrated heat enthalpies as determined by peak integration of the ITC heat flow signal (B), where the molar ratio = HSA:MEDI7219. [HSA] = 0.15 mM, [MEDI7219] = 2.5 mM. Buffer composition: 8.2 mM potassium phosphate monobasic, 1.8 mM potassium phosphate dibasic, 140 mM NaCl.

Second, MEDI7219 was titrated into HSA in the presence of polysorbate. For these experiments, a sample graph depicting MEDI7219 being titrated into HSA in the presence of RG T20 is shown in Figure 5.

**Figure 5:**
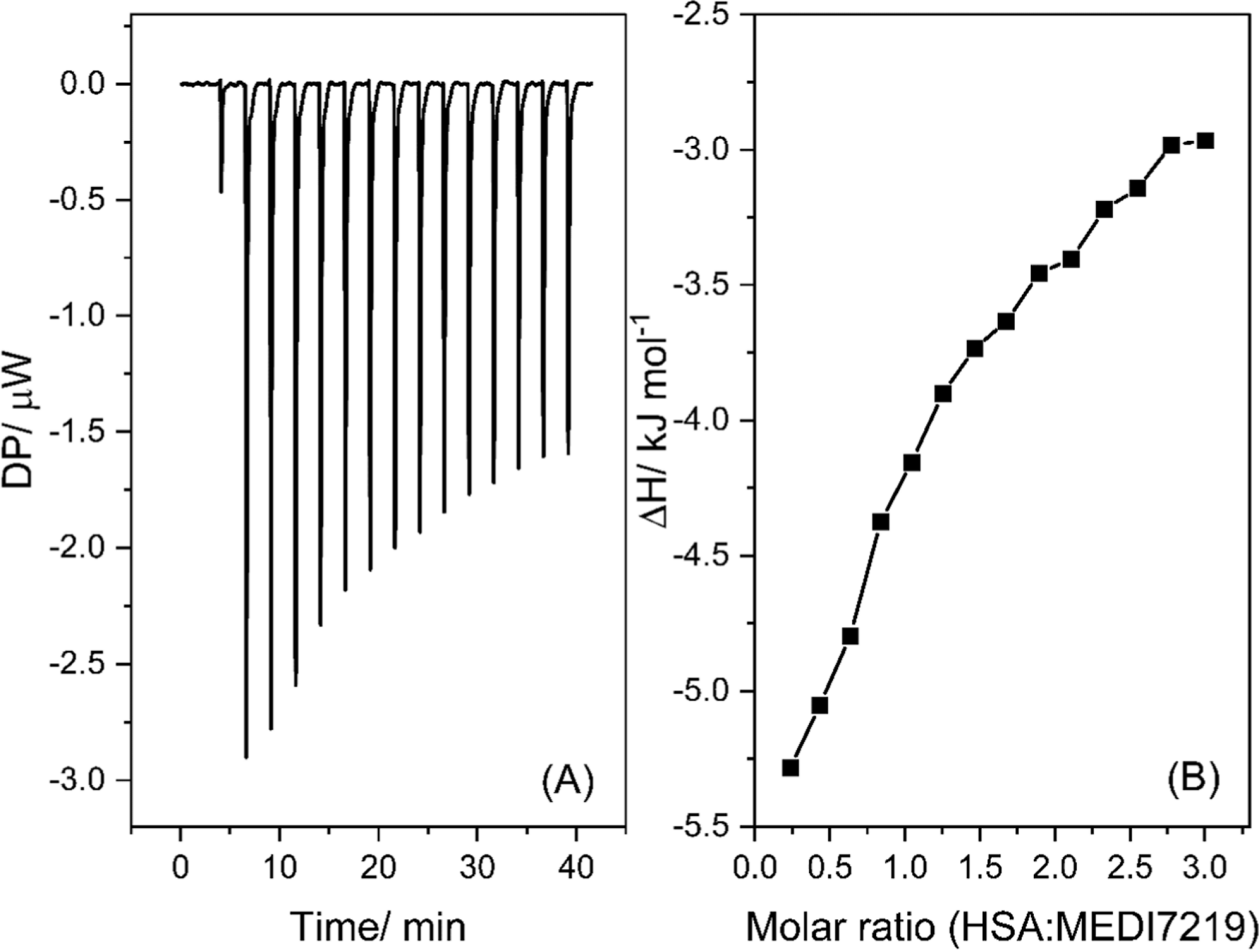
ITC thermogram of the interaction between MEDI7219 and HSA in the presence of RG T20 at 30 °C. Heat flow signal (A). Integrated heat enthalpies as determined by peak integration of the ITC heat flow signal (B), where the molar ratio = HSA:MEDI7219. [HSA] = 0.15 mM, [MEDI7219] = 2.5 mM. Polysorbate concentration = 1 × CMC of each polysorbate.^19^ Buffer composition: 8.2 mM potassium phosphate monobasic, 1.8 mM potassium phosphate dibasic, 140 mM NaCl.

Overall, ITC facilitated calculation of the thermodynamic binding parameters summarized in Table 1.

**Table 1:** Thermodynamic Binding Parameters of MEDI7219 Interactions with HSA in the Absence and Presence of Polysorbate RG and Super Refined (SR)

binding interaction	dissociation constant (M)	Δ*G* (kJ mol^–1^)	Δ*H* (kJ mol^–1^)	–*T*Δ*S* (kJ mol^–1^)
MEDI7219 → HSA^a^	4.03 × 10^–5^ (±1.52 × 10^–5^)	–25.5 (±0.97)	35.4 (±8.19)	–60.9 (±7.70)
MEDI7219 → HSA + RG T20	1.76 × 10^–4^ (±1.11 × 10^–4^)	–21.8 (±0.87)	–6.6 (±3.20)	–15.3 (±1.28)
MEDI7219 → HSA + SR T20	5.56 × 10^–5^ (±7.53 × 10^–6^)	–24.7 (±0.97)	27.8 (±9.32)	–52.5 (±4.35)
MEDI7219 → HSA + RG T80	2.40 × 10^–5^ (±1.67 × 10^–6^)	–26.8 (±1.10)	25.3 (±0.70)	–52.1 (±4.34)
MEDI7219 → HSA + SR T80	3.51 × 10^–5^ (±3.07 × 10^–6^)	–25.9 (±1.04)	25.4 (±1.03)	–51.3 (±6.16)

a Values in this row are stated as averages across all experiments although each experiment pathway provided unique values for individual calculation of each α value. *N* = 3. Error = ± SD.

When considering the influence of RG T20 on MEDI7219–HSA interactions, it can be seen that RG T20 was the only polysorbate that produced an exothermic heat profile with an Δ*H* of −6.6 kJ mol^–1^. This could be due to the binding activity between RG T20 and HSA, as the polysorbate is already present, the polysorbate could have a larger or smaller binding affinity than the other polysorbates which changes the consequent binding of MEDI7219. This is shown in Table 1 as the dissociation constant, whereby a smaller dissociation constant suggests a higher binding affinity. Another explanation could be that RG T20 performs differently in its ability to protect HSA molecules from interfacial damage, resulting in exothermic heat enthalpies as MEDI7219 and HSA undergo changes in their structures not possible when other polysorbates are present. On the other hand, the influence of SR T20 gave rise to a different effect in that SR T20 only caused a slight increase in the dissociation constant, this being a binding dissociation constant of 5.56 × 10^–5^ M, with a similar Δ*G* value of −24.7 kJ mol^–1^, i.e., the interaction still occurred spontaneously. Table 2 presents the α term for each experiment,^15^ this value was derived by taking the reciprocal of each dissociation constant to produce the association constant, then dividing the association constant of MEDI7219 being titrated into HSA in the presence of polysorbates (*K*_B/A_) by the association constant of MEDI7219 being titrated into HSA (*K*_B_). The value for *K*_B/A_/*K*_B_ shall be denoted as α_i_.

**Table 2:** Calculated α_i_ Value for MEDI7219–HSA Interactions in the Presence of Different Polysorbate Molecules (*K*_B/A_/*K*_B_)^15^ ^a^

binding interaction	α_i_
MEDI7219 → HSA + RG T20	0.37
MEDI7219 → HSA + SR T20	0.40
MEDI7219 → HSA + RG T80	1.68
MEDI7219 → HSA + SR T80	1.30

a [HSA] = 0.15 mM, [MEDI7219] = 2.5 mM. Polysorbate concentration is 1 × CMC of each polysorbate.^19^ Buffer composition: 8.2 mM potassium phosphate monobasic, 1.8 mM potassium phosphate dibasic, 140 mM NaCl.

For RG and SR T20, the α value was below 1. According to Velazquez-Campoy and co-workers, this suggests that the formation of the ternary complex was not thermodynamically favorable when compared with independent binding of each ligand. This is known as negative cooperativity.^15^ Next, considering the influence of T80, Table 1 demonstrates that RG T80 produced an endothermic heat flow signal and an Δ*G* of −26.8 kJ mol^–1^. The binding of MEDI7219 was just as likely to take place in the presence of RG T80 as its absence. RG T80 decreased the dissociation constant of MEDI7219 by 2-fold to 2.40 × 10^–5^ M. This type of interaction was also observed in the presence of SR T80 with a similar Δ*G* of −25.9 kJ mol^–1^ and a decreased dissociation constant, although the decrease was smaller than that of RG T80 (3.51 × 10^–5^ M). The α values for RG T80 and SR T80 were 1.68 and 1.30, respectively; this suggests that the resulting ternary complex was thermodynamically more favorable than if both ligands were to bind independently, this is known as positive cooperativity. While the α values for RG T80 and SR T80 are different, it is not possible to determine if this difference is significant as there are only two values but it is a possible avenue for future investigation.

The differences in the binding interactions induced by T20 and T80 could be due to their differing structures. T20 possesses a hydrophobic chain consisting of a lauric acid component which is shorter in length than T80, which possesses an oleic acid component.^22^ As seen in similar studies, this could have affected the nature of the interactions that occurred.^23,24^ For example, the presence of T80 may have led to changes in the shape of HSA which exposed groups in HSA that could then bind to MEDI7219 with a higher affinity.

While the influence of polysorbate molecules on HSA–MEDI7219 interactions has been considered, it would also be appropriate to consider the influence of MEDI7219 on HSA–polysorbate interactions. Figures 6 and 7 are examples of polysorbate–HSA interactions; specifically, they depict RG T20 interactions in the absence and presence of MEDI7219. All polysorbates produced exothermic heat flow signals and were similar to previous experiments carried out by this research group.^25^

**Figure 6:**
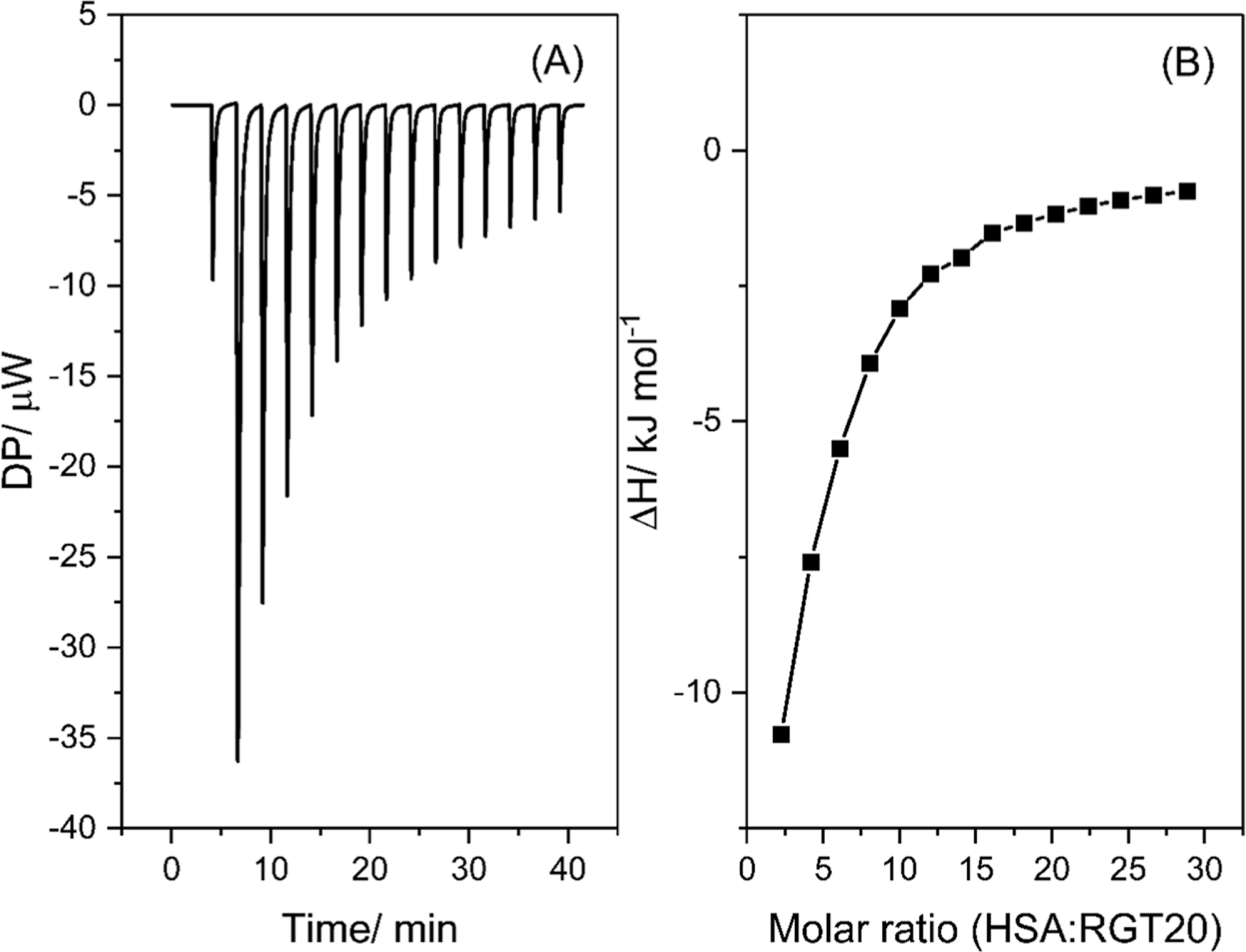
Example of an ITC thermogram of the interaction between RG T20 and HSA at 30 °C. Heat flow signal (A). Integrated heat enthalpies as determined by peak integration of the ITC heat flow signal (B), where the molar ratio = HSA:RG T20. [HSA] = 0.15 mM and polysorbate concentration = 1 × CMC.^19^ Buffer composition: 8.2 mM potassium phosphate monobasic, 1.8 mM potassium phosphate dibasic, 140 mM NaCl.

**Figure 7:**
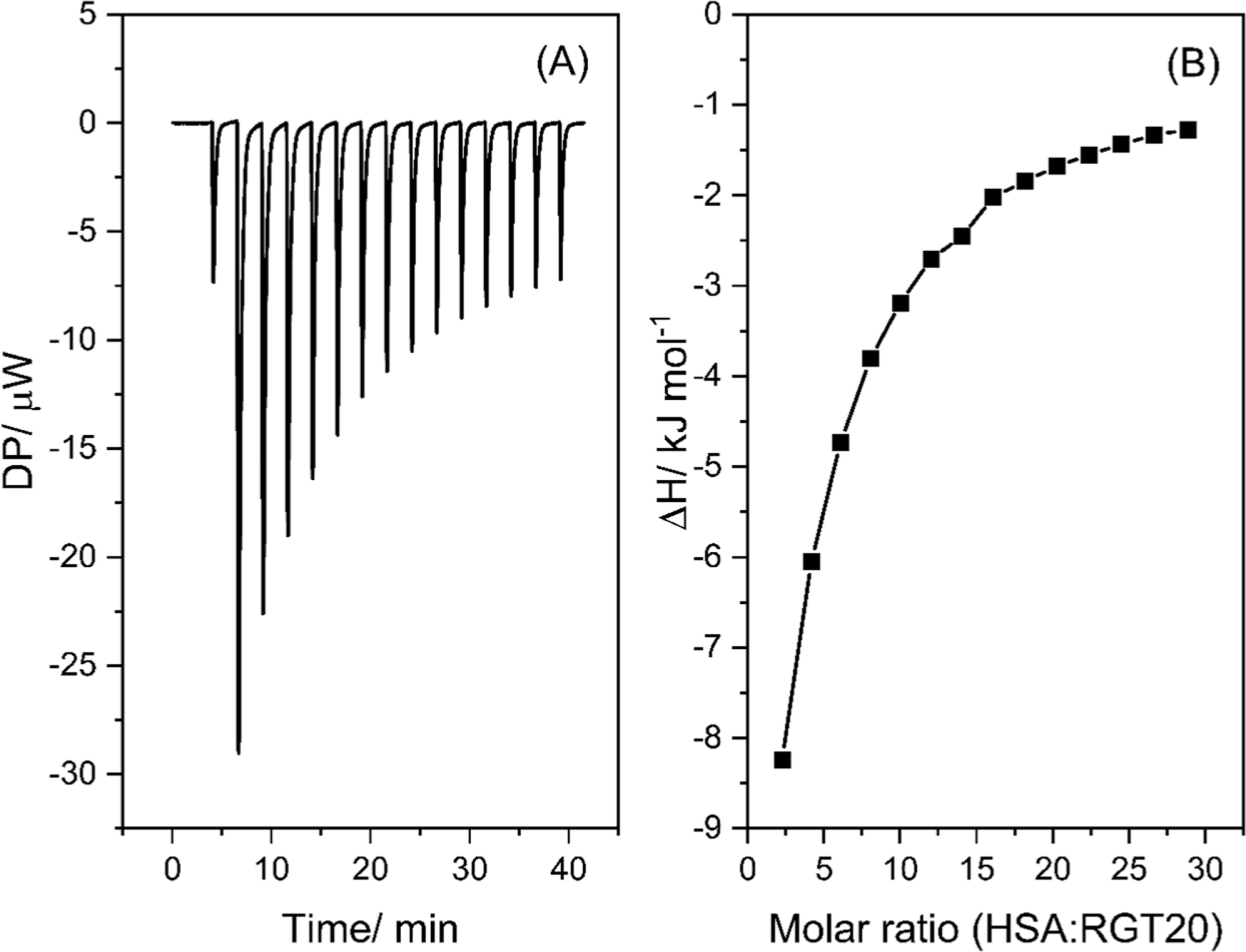
Example of an ITC thermogram of the interaction between RG T20 HSA in the presence of MEDI7219 at 30 °C. Heat flow signal (A). Integrated heat enthalpies as determined by peak integration of the ITC heat flow signal (B), where the molar ratio = HSA:RG T20. [HSA] = 0.15 mM, polysorbate concentration is 1 × CMC of each polysorbate.^19^ [MEDI7219] = 2.5 mM. Buffer composition: 8.2 mM potassium phosphate monobasic, 1.8 mM potassium phosphate dibasic, 140 mM NaCl.

Table 3 presents the calculated thermodynamic properties of the binding interactions between polysorbates and HSA in the presence of MEDI7219. For each surfactant investigated, it can be seen that the presence of MEDI7219 reduced the binding affinity, Δ*G* and Δ*H*. Changes in entropy in all cases were comparatively small compared with changes in Δ*H*, indicating the reaction was not entropically driven. This suggests that when MEDI7219 was present in a solution, it reduced the likelihood of polysorbate interacting with HSA and, if an interaction did take place, the strength of that binding was reduced.

**Table 3:** Thermodynamic Binding Parameters of Polysorbate Interactions with HSA in the Absence and Presence of MEDI7219^a^

binding interaction	dissociation constant (M)	Δ*G* (kJ mol^–1^)	Δ*H* (kJ mol^–1^)	–*T*Δ*S* (kJ mol^–1^)
RG T20 → has	1.03 × 10^–3^ (±8.91 × 10^–7^)	–17.30 (±0.87)	–83.70 (±36.7)	66.40 (±2.74)
RG T20 → HSA + MEDI7219	2.21 × 10^–3^ (±5.57 × 10^–8^)	–15.40 (±0.77)	–9.07 (±9.4 × 10^–7^)	–6.36 (±0.26)
SR T20 → has	7.21 × 10^–4^ (±4.30 × 10^–5^)	–18.20 (±0.91)	–43.10 (±6.15)	24.90 (±1.03)
SR T20 → HSA + MEDI7219	1.69 × 10^–3^ (±3.52 × 10^–4^)	–16.10 (±0.81)	–4.19 (±0.62)	–11.90 (±0.50)
RG T80 → has	1.01 × 10^–3^ (±1.11 × 10^–4^)	–17.40 (±0.87)	–9.13 (±1.29)	–8.26 (±0.34)
RG T80 → HSA + MEDI7219	1.76 × 10^–3^ (±5.33 × 10^–4^)	–16.00 (±0.80)	–8.75 (±4.46)	–7.25 (±0.23)
SR T80 → has	5.13 × 10^–4^ (±1.4 × 10^–5^)	–19.10 (±0.10)	–30.10 (±2.02)	11.00 (±0.45)
SR T80 → HSA + MEDI7219	1.28 × 10^–3^ (±9.14 × 10^–6^)	–16.80 (±0.84)	–8.72 (±2.7 × 10^–5^)	–8.09 (±0.33)

a [HSA] = 0.15 mM, [MEDI7219] = 2.5 mM. Polysorbate concentration = 1 × CMC of each polysorbate.^19^ Buffer composition: 8.2 mM potassium phosphate monobasic, 1.8 mM potassium phosphate dibasic, 140 mM NaCl. *N* = 3. Error = ± SD.

The results calculated in Table 3 facilitated calculation of α values, as displayed in Table 4. The α values were derived by taking the reciprocal of each dissociation constant to produce the association constant and then dividing the association constant of polysorbate being titrated into HSA in the presence of MEDI7219 (*K*_A/B_) by the association constant of polysorbate being titrated into HSA (*K*_A_). The values for *K*_A/B_/*K*_A_ are denoted as α_ii_.

**Table 4:** Calculated α_ii_ Values of Polysorbate–HSA Interactions in the Presence of MEDI7219 (*K*_A/B_/*K*_A_)^15^ ^a^

binding interaction	α_ii_
RG T20 → HSA + MEDI7219	0.47
SR T20 → HSA + MEDI7219	0.43
RG T80 → HSA + MEDI7219	0.57
SR T80 → HSA + MEDI7219	0.40

a [HSA] = 0.15 mM, [MEDI7219] = 2.5 mM. Polysorbate concentration is 1 × CMC of each polysorbate.^19^ Buffer composition: 8.2 mM potassium phosphate monobasic, 1.8 mM potassium phosphate dibasic, 140 mM NaCl.

The values displayed in Table 4 all indicate negative cooperativity between MEDI7219 and both grades of T20 and T80. Velazquez-Campoy and co-workers propose that α_i_ should equal α_ii_ independent of which reaction pathway is taken as this preserves the mass conservation principle.^15^ However, in these experiments, α_i_ does not equal α_ii_; this could be due to two fundamentally different interactions taking place, which may be influenced by the order in which the ligands are added, leading to changes in the composition of the final adduct. Such information is incredibly valuable to formulators when creating ternary biologic-based systems such as this.

### 3.2 DSC

The thermal stability of a molecule and its interactions can play an important role in determining the long-term stability of a product, for example, if the thermal stability of a molecule increases with the addition of an excipient, it would be reasonable to assume that the long-term stability at typical storage conditions has also increased.^16^ When investigating the thermal stability of proteins using DSC, the transition between the native state of the protein and a denatured state is usually seen at the midpoint of a thermal transition peak, known as the *T*_m_. It should be noted that MEDI7219 is a peptide with no secondary structure and therefore cannot denature via unfolding and does not produce a measurable DSC response. Consequently, all DSC profiles in this study focus on changes in the thermal stability of HSA. First, it was found that when HSA was heated in the presence of MEDI7219, the thermal profile of HSA changed. Figure 8 displays examples of the DSC profiles for HSA and the HSA–MEDI7219 complex.

**Figure 8:**
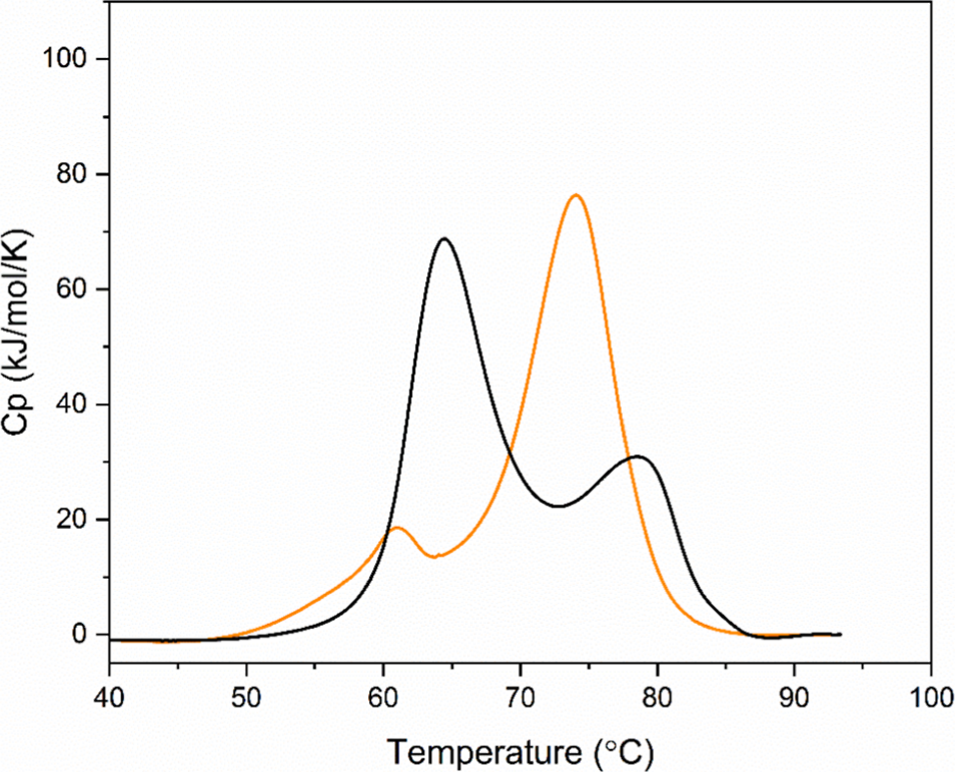
DSC profiles for HSA (black) and HSA–MEDI7219 (orange).

From Figure 8, it can be seen that without any additional molecules present, HSA displayed two distinct events, a large event with a *T*_m_ of 63.9 °C and another smaller event with a *T*_m_ of 76.3 °C. This indicates at least two different thermal events involving different protein domains within HSA. When next considering the effect of adding MEDI7219, it can be observed that there was a change in the thermal profile, primarily a change in the relative size of each event. The DSC profile for the HSA–MEDI7219 complex displayed a small event at a slightly lower temperature than HSA without MEDI7219, at around 61.3 °C. However, the total area for this peak was greatly reduced in comparison; the second event also occurred at the slightly lower temperature of 74.0 °C (Table 5).

**Table 5:** DSC Results for HSA and HSA–MEDI7219^a^

protein	ligand	total area (kJ/mol)	*T*_m_ 1 (°C)	*T*_m_ 2 (°C)
HSA	none	722 (±43.5)	63.9 (±1.2)	76.3 (±0.1)
HSA	MEDI7219	806.3 (±5.8)	61.3 (±0.4)	74.0 (±0.1)

a Number of experimental repeats ≥3, error = ± SD.

When looking at the impact of the addition of MEDI7219 in Figure 8, it is clear that there was a decrease in the area of the first peak and an increase in the area of the second peak, producing an overall increase in the total area (Table 5). However, the authors propose that the first peak of HSA without MEDI7219 appearing at 63.9 °C, has been stabilized by its interaction with MEDI7219. This results in an increase in its *T*_m_ from 63.9 °C up to around 74 °C. This shifted peak then overlaps with the second peak which had an initial *T*_m_ of 76.3 °C and produces the peak which appears with a *T*_m_ of 74 °C. This explanation is offered as HSA is a multidomain protein, specifically it possesses three domains that are available for binding.^26^ One of these binding sites could have become bound to MEDI7219, increasing that domain’s thermal stability and revealing a third peak that can be seen at 61.3 °C. This is a unique finding and suggests that HSA-GLP-1 receptor agonist complexes are more thermally stable when compared with HSA without the bound peptide.

Second, analysis of the HSA–MEDI7219 complex with polysorbates was performed, as displayed in Figures 9 and 10.

**Figure 9:**
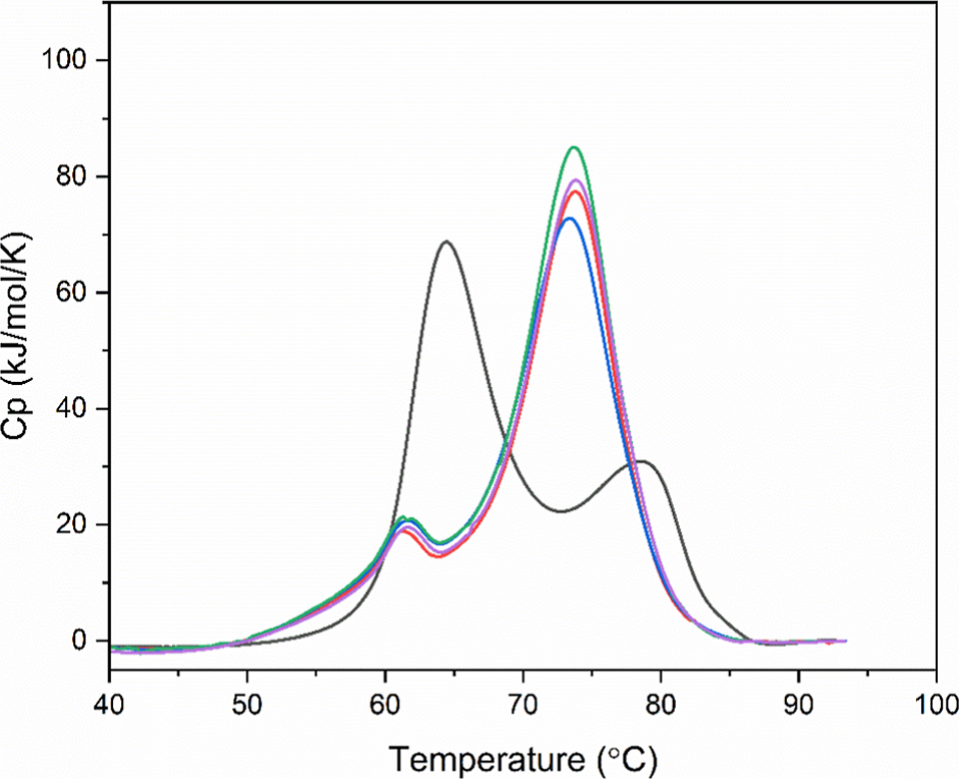
Examples of DSC profiles for MEDI7219–polysorbate–HSA interactions at 0.1 × CMC. HSA without MEDI7219 and polysorbate (black) HSA with MEDI7219 and RG T20 (red), SR T20 (blue), RG T80 (green), and SR T80 (purple).

**Figure 10:**
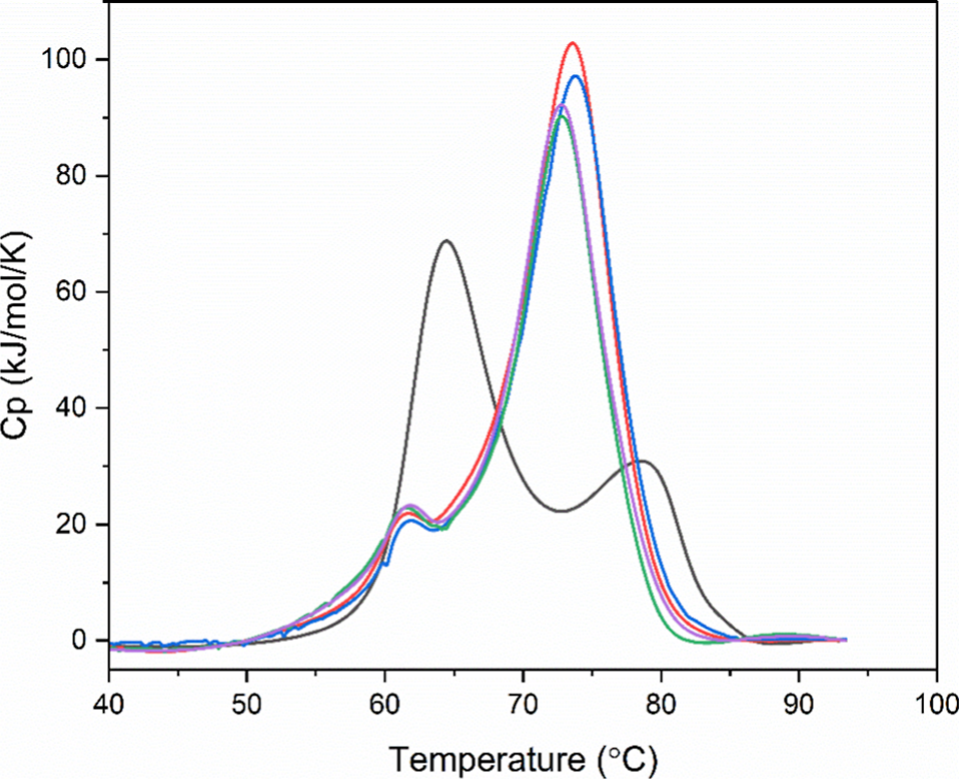
Examples of DSC profiles for MEDI7219–polysorbate–HSA interactions at 1 × CMC. HSA without MEDI7219 and polysorbate (black) HSA with MEDI7219 and RG T20 (red), SR T20 (blue), RG T80 (green), and SR T80 (purple).

At 0.1 × CMC all polysorbates investigated exhibited a small event with *T*_m_ values around 61 °C (Table 6) and a larger event with *T*_m_ values around 75 °C. Previous studies have established that HSA interactions with polysorbates produced similar DSC events to the ones seen in Figure 9 (∼64 °C for the first event and between 71 and 77 °C for the second^25^). The DSC profiles indicated that all polysorbates studied increased the thermal stability of HSA to a similar extent. This is supported by previous literature which has showed that RG polysorbate 20 and 80 can increase the *T*_m_ of HSA.^23^ Table 6 also indicates that the total area of each DSC peak was greater for HSA in the presence of all polysorbate grades than that for HSA without additional components. This increase in area was directly proportional to an increase in available HSA molecules that then underwent a thermal unfolding event,^27^ implying increased stability of HSA.

**Table 6:** DSC Results for HSA and HSA–MEDI7219–Polysorbates at 0.1 × CMC^a^

protein	ligand	total area (kJ/mol)	*T*_m_ 1 (°C)	*T*_m_ 2 (°C)
HSA	none	722 (±43.5)	63.9 (±1.2)	76.3 (±0.1)
HSA	RG T20 + MEDI7219	797 (±18.0)	61.3 (±0.0)	73.9 (±0.1)
HSA	SR T20 + MEDI7219	839 (±28.0)	61.7 (±0.1)	73.4 (±0.0)
HSA	RG T80 + MEDI7219	914.5 (±21.5)	61.6 (±0.0)	74.1 (±0.4)
HSA	SR T80 + MEDI7219	877.0 (±9.0)	61.2 (±0.1)	73.9 (±0.1)

a Number of experimental repeats ≥2, error = ± SD.

At polysorbate concentrations of 1 × the CMC, the *T*_m_ values are similar to the values at 0.1 × CMC. The DSC events again showed that for all polysorbates investigated, two distinct peaks with *T*_m_ values of around 62 and 74 °C were seen (Figure 10).

A summary of the values obtained from DSC analysis for polysorbate concentrations at 1× CMC is displayed in Table 7.

**Table 7:** DSC Results for HSA and HSA–MEDI7219–Polysorbates at 1× the CMC^a^

protein	ligand	total area (kJ/mol)	*T*_m_ 1 (°C)	*T*_m_ 2 (°C)
HSA	none	722 (±43.5)	63.9 (±1.2)	76.3 (±0.1)
HSA	RG T20 + MEDI7219	1005 (±5.0)	61.7 (±0.1)	74.0 (±0.5)
HSA	SR T20 + MEDI7219	1095 (±5.0)	61.8 (±0.3)	75.0 (±1.2)
HSA	RG T80 + MEDI7219	947.0 (±15.0)	61.7 (±0.1)	72.8 (±0.0)
HSA	SR T80 + MEDI7219	977.0 (±43)	62.0 (±0.2)	72.8 (±0.4)

a Number of experimental repeats ≥2, error = ± SD.

Although the *T*_m_ values are similar in the presence of polysorbates, the total area of the DSC events increased by an average of 39% (approximately 200 kJ/mol) (Table 7). This increase implies that there were more native HSA molecules available to undergo thermal transitions. This trend suggests that by increasing the polysorbate concentration (and keeping the concentration of MEDI7219 constant), a formulation will be improved through more of the native molecules being present. Therefore, it would be logical to predict that higher concentrations of polysorbates would continue to increase the total area of the DSC events. However, higher concentrations could not be used (>1 × the CMC) for ITC experiments due to the dilution effect of injection, requiring a solution to be prepared at 5 times the intended concentration, i.e., above the solubility limit of each polysorbate.

From Figure 10, with *T*_m_ values similar to those seen in the DSC events for HSA–MEDI7219 without polysorbate, it appeared that polysorbates did not improve the thermal stability of HSA any more than adding MEDI7219. Therefore, the protein–peptide complex was already highly thermally stable. The ability for a peptide to stabilize a protein is not completely unexpected and similar behavior can be seen in biosurfactants.^28^ Biosurfactants, similar to chemical polysorbates, possess the ability to reduce surface tension and also exhibit the ability to spontaneously organize into structures such as micelles.^29^ Unlike polysorbates, biosurfactants are extracted from natural sources or, in the case of peptide-based biosurfactants, are designed using amino acids. MEDI7219 contains two lipid chains, and it could be this property that allowed MEDI7219 to act like a biosurfactant and stabilize HSA. This hypothesis further suggests that while polysorbates may not increase intramolecular thermal stability, they instead significantly increased the enthalpy of the DSC events and therefore increased the concentration of available HSA. This effect could be due to the ability of polysorbates to protect against the interfacial aggregation of proteins, leading to an increase in intermolecular thermal stability when compared with experiments without polysorbate present.^30^

## 4 Conclusions

ITC experiments showed that the presence of both RG and SR T20 decreased the binding affinity of the peptide MEDI7219 to HSA, and this may be useful if the desired outcome for the patient is to administer multiple biologics without interaction between them. On the other hand, both RG and SR T80 increased the binding affinity of MEDI7219 to ; this may be useful if a biologic is developed that requires a carrier to maintain stability in vivo. DSC demonstrated that when HSA was bound to MEDI7291, it became more thermally stable, yet the stability of HSA did not increase further when polysorbate molecules were added to the formulation. This could be due to the fact that MEDI7219 binds with a higher affinity than polysorbate molecules, and therefore MEDI7219 binds first and possibly prevents further binding of the polysorbate molecules. This study highlights the use of ITC and DSC as insightful methods to build a fundamental understanding of the behaviors of biologics in the presence of other species. In conclusion, investigating the influence of commonly used excipients on protein–peptide interactions provides insight into the most compatible combinations of molecules for coformulations.
